# Plant-Derived Natural Products and Their Biomedical Properties: Recent Advances and Future Directions

**DOI:** 10.3390/life13102105

**Published:** 2023-10-23

**Authors:** Charalampia Amerikanou, Efstathia Papada

**Affiliations:** 1 Department of Nutrition and Dietetics, School of Health Science and Education, Harokopio University, 17671 Athens, Greece; camer@hua.gr; 2 Division of Medicine, University College London, London WC1E 6JF, UK

Nature has always been a source of inspiration and innovation to humanity. Throughout history, plants have provided us with not only sustenance but also a vast array of medicines, materials, and resources. In recent decades, there has been intense research interest in harnessing the biomedical potential of plant-derived natural products. To this end, the evolution of several methods for the extraction, isolation, and chemical characterization of plant-derived natural products has substantially contributed to the identification of their potential therapeutic properties. In this Special Issue, we explore recent advances in plant-derived natural products and their biomedical properties via intriguing in vitro and in vivo research articles and literature reviews.

Recently, Lantzouraki et al. showed that the extracts of two *Artemisia* species, *Artemisia arborescens* and *Artemisia inculta Delile*, from the Greek island of Crete exhibit an interesting profile of secondary metabolites [1]. More specifically, a high phenolic and terpenoid content and safe metal levels were reported in the aqueous–glycerolic extract of these two species. Furthermore, this extract offered a higher antioxidant potential compared to decoctions and methanolic extracts, suggesting its potential use not only in the food industry but also for dermocosmetic and pharmacological applications, given that glycerol increases the transdermal delivery of active substances [1]. On a similar note, the chemical compositions of different extracts of another Mediterranean plant have been evaluated. Ouahabi et al. determined the fatty acid content in the n-hexane extract and the phenolic content in the methanolic extract of the leaves of Moroccan *Pistacia lentiscus* (mastic tree), with the main component being linoleic acid in the first case and catechin in the second [2]. The extracts also exhibited significant antioxidant, antimicrobial, and antifungal activity, with in silico analyses revealing some interesting interactions between the identified components of the extracts and specific enzymes. An example interaction is between catechin and enoyl-acyl carrier protein reductase (FabI), a native *E. coli* enzyme, with potential inhibitory activity, supporting the potential use of mastic tree and its extracts in drug development [2]. *Euonymus laxiflorus* Champ. (ELC), a Vietnamese medicinal plant already known for its antioxidant and antidiabetic activity, has been investigated for its anti-acetylcholinesterase effect, an important mechanism for the management of Alzheimer’s disease [3]. Interestingly, the ELC trunk bark extract featured a high phenolic and flavonoid content, and its in vitro anti-acetylcholinesterase activity was comparable to that of berberine chloride, a known acetylcholinesterase inhibitor. The compounds with the highest concentration included chlorogenic acid, epigallocatechin gallate, epicatechin, apigetrin, and quercetin, while docking-based simulations revealed their potential drug properties due to their significant binding energy to acetylcholinesterase [3].

Several animal studies have also emerged regarding the exploitation of the biomedical properties of plant-derived natural products. Recently, a rat model of diet-induced obesity was used to explore the effect of alcoholic and aqueous extracts of *Jatropha tanjorensis* (JT) and *Fraxinus micrantha* (FM) leaves on parameters related to metabolic syndrome [4]. A reduction in weight gain, food intake, serum glucose, and lipid profile was observed in rats treated with the extracts compared to the control high-fat-fed rats. Interestingly, the levels of antioxidant enzymes increased significantly after treatment with JT extract and showed similar levels to those achieved after treatment with orlistat, a common medicine used in obesity management [4]. Another study on rats investigated the gastroprotective effects of *Eurycoma longifolia* Jack (ELJ), a popular traditional herbal medicine of Southeast Asia [5]. The oral administration of a high dose of ELJ (1200 mg/kg) in carrageenan-induced rat paw edema showed a similar anti-antinociceptive and anti-inflammatory activity to aspirin (300 mg/kg) and reduced the rectal temperature of yeast-infected rats, suggesting central antipyretic effects. Finally, in the same animal model, treated with acidified ethanol for the development of gastric ulcers, different ELJ extract doses prevented gastric ulcer formation, demonstrating its gastroprotective properties [5]. 

*Ocimum sanctum* (Tulsi) is a well-known medicinal herb, commonly used in the Ayurvedic system for anxiety, cough, diarrhea, fever, vomiting, and other ailments [6]. Yadav et al. explored whether its extract administration to mice with a photothrombotic-ischemic-stroke-like injury could show an effect on their brain and the lipidomic profile of the brain and plasma. Untargeted lipidomic profiling with the Q-Exactive Mass Spectrometer revealed 77 upregulated and 33 downregulated lipid species in the brains of untreated lesioned mice compared to those treated with Tulsi. The most interesting finding was the increased presence of lysophosphatidylcholine (LPC) (16:1) in the brain of Tulsi-treated mice. This lysophospholipid is a major component of membranes, which implicates it in immune regulation after cerebral ischemia in rats, and it has also been found in Tulsi extract, suggesting a neuroprotective effect [6]. Another study evaluated the anticarcinogenic effects of hesperetin (HES), a naturally occurring flavone in *Citrus aurantium* L. (Rutaceae) fruit peel, alone and in combination with capecitabine (CAP), a chemotherapeutic drug, on 1,2 dimethylhydrazine (DMH)-induced colon carcinogenesis in Wistar rats [7]. The results showed that treatment with HES and/or CAP prevented histological cancerous changes in combination with a decrease in colon-Ki67 expression and serum carcinoembryonic antigen. Additionally, treatments with HES and/or CAP induced a significant reduction in serum lipid peroxides, and an increase in serum reduced glutathione, as well as the enhancement of colon-tissue superoxide dismutase, glutathione reductase, and glutathione-S-transferase activities. Interestingly, an increase in the mRNA expression of the anti-inflammatory IL-4, as well as the proapoptotic protein p53, in the colon tissues of the DMH-administered rats treated with HES and/or CAP was reported [7].

The therapeutic effects of aqueous extract (BCAE) and ethanolic extract (BCEE) obtained from the aerial parts of *Brocchia cinerea* (Delile) were evaluated by Agour and colleagues [8]. Firstly, the total polyphenol content (TPC), total flavonoid content (TF), and condensed tannin content (CT) were determined, and the results showed that BCEE had the highest content of polyphenols, flavonoids, and tannins. In vitro, DPPH, FRAP, and TAC were used to evaluate antioxidant efficacy, and BCEE demonstrated a strong antiradical activity against DPPH and a medium iron-reducing potential, while BCAE inhibited the growth of the antibiotic-resistant bacterium *P. aeruginosa.* The analgesic power was evaluated in vivo using the abdominal contortion model in mice, and the anti-inflammatory activity was assessed via the carrageenan-induced edema model in rats, while wound healing was evaluated in an experimental second-degree-burn model. BCAE exhibited significant pharmacological effects and analgesic efficacy and also contributed to the re-epithelialization of wounds [8]. Salamatullah investigated the chemical composition, as well as the antioxidant, anti-inflammatory, and analgesic properties, of a polyphenol-rich fraction from *Withania adpressa* Coss. ex Batt [9]. High-performance liquid chromatography (HPLC) was applied and revealed bioactive phenols including epicatechin, apigenin, luteolin, quercetin, caffeic acid, p-coumaric acid, and rosmarinic acid. This fraction showed anti-free-radical potency and a good total antioxidant capacity. In a rat model, the polyphenol-rich fraction strongly alleviated the inflammatory effect of carrageenan injected into the plantar fascia of rats and showed a good analgesic effect after heat stimulation.

Apart from the new research in vitro, and in vivo in animal models, several researchers around the world have attempted, through literature reviews, to gather and critically evaluate the current evidence on the biomedical properties of various plant-derived natural compounds. A recent review evaluated plagiochilins, a series of seco-aromadendrane-type sesquiterpenes isolated from leafy liverworts of the genus *Plagiochila* [10]. Currently, a total of 24 plagiochilins and many derivatives have been isolated and characterized, but there are limited studies on their pharmacological properties. Interestingly, plagiochilins A and C have demonstrated notable antiproliferative activity against cultured cancer cells by inhibiting the termination phase of cytokinesis. However, these compounds should be further evaluated for their antiproliferative potential [10]. Another recent review compiled the evidence regarding the biomedical properties of mogrol, a triterpene found in the fruits of the traditional Chinese medicinal plant *Siraitia grosvenorii*, and also highlighted the current research gaps and pointed out directions for future work [11]. Research has shown that mogrol could act as a therapeutic candidate with multiple pharmacological properties, including, but not limited to, neuroprotective, anticancer, anti-inflammatory, and antidiabetic effects. Although the molecular mechanisms behind these activities might involve several important targets, including AMPK, TNF-α, and NF-κB, there is still a gap in the literature concerning the exact mechanisms and targets [11].

*Juglans regia* Linn., a tree accounting for around 88% of the total walnut production globally, has been evaluated for the phytochemical content of its various components, including the bark, leaves, and fruit. Bhat and colleagues reviewed the scientific literature on the antimicrobial, antioxidant, antifungal, and anticancer properties of various compounds isolated from different solvents and different parts of *J. regia* and suggested that synthetic analogues and various extracts should be further assessed in a concentration-dependent manner to increase our understanding of these promising properties [12]. Last but not least, Davila and Papada critically evaluate the relevant evidence around the use of plant-derived natural products in the management of inflammatory bowel disease, with a specific focus on the clinical evidence so far for curcumin, Mastiha, *Boswellia serrata*, and *Artemisia absinthium*. As the results from human trials with relatively small sample sizes are very limited, the authors highlight the need to prioritize clinical trials focusing on phytochemical bioavailability, optimal doses, and safety data, which are essential for the inclusion of plant-derived natural compounds in the management pathways of chronic inflammatory conditions [13].

In conclusion, the research findings in this Special Issue, summarized above, shed further light on our knowledge and understanding of the effects of various plant-derived natural compounds on health and disease. In order for these compounds to be considered for use in established therapeutic regimes for chronic and acute pathological conditions, it is crucial to gain further insights into their exact mechanisms of action, interactions with drugs and nutrients, dosages, and safety considerations.
